# Triple repeated fetal congenital heart disease linked to *PLD1* mutation: a case report

**DOI:** 10.1186/s13256-023-04149-9

**Published:** 2023-09-29

**Authors:** Yuki Masuda, Yoko Nagayasu, Hikaru Murakami, Ruri Nishie, Natsuko Morita, Sosuke Hashida, Atsushi Daimon, Misa Nunode, Hiroshi Maruoka, Masae Yoo, Takumi Sano, Yutaka Odanaka, Satoe Fujiwara, Daisuke Fujita, Nobuhiko Okamoto, Masahide Ohmichi

**Affiliations:** 1 Department of Obstetrics and Gynecology, Osaka Medical and Pharmaceutical University, Takatsuki, Japan; 2 Department of Obstetrics and Gynecology, Saiseikai Suita Hospital, Suita, Japan; 3 Department of Pediatrics, Osaka Medical and Pharmaceutical University, Takatsuki, Japan; 4 Department of Medical Genetics, Osaka Women’s and Children’s Hospital, Izumi, Japan

**Keywords:** Congenital heart disease, Cardiology, Genetic, Genetic disease, *PLD1*

## Abstract

**Background:**

Congenital heart disease occurs in approximately 1 in 100 cases. Although sibling occurrence is high (3–9%), the causative genes for this disease are still being elucidated. *PLD1* (*Phospholipase D1*) is a recently discovered gene; however, few case reports have been published on it. In this report, we describe a case of triplicate fetal congenital heart disease that was diagnosed as a PDL1 mutation. Our objective is to explore the clinical manifestations of *PLD1* mutations in this particular case.

**Case presentation:**

A 32-year-old Japanese woman (gravida, para 0) was introduced since fetus four chamber view was not clear and was diagnosed with ductus arteriosus-dependent left ventricular single ventricle and pulmonary atresia at 21 weeks and 1 day of gestation during her first pregnancy. Artificial abortion using Gemeprost was performed at 21 weeks and 5 days of gestation. The second pregnancy was diagnosed as pulmonary atresia with intact ventricular septum with cardiomegaly, a cardiothoracic area ratio of more than 35%, and a circulatory shunt at 13 weeks and 3 days of gestation. Subsequently, intrauterine fetal death was confirmed at 14 weeks and 3 days of gestation. Regarding the third pregnancy, fetal ultrasonography at 11 weeks and 5 days of gestation showed mild fetal hydrops and moderate tricuspid valve regurgitation. At 16 weeks and 5 days of gestation, the fetus was suspected to have a left ventricular-type single ventricle, trace right ventricle, pulmonary atresia with intact ventricular septum, or cardiomyopathy. Cardiac function gradually declined at 26 weeks of gestation, and intrauterine fetal death was confirmed at 27 weeks and 5 days of gestation. The fourth pregnancy resulted in a normal heart with good progression and no abnormal baby. We submitted the first and second fetuses’ umbilical cord, third fetus’ placenta, and the fourth fetus’ blood to genetic testing using whole exome analysis with next generation sequencing. Genetic analysis identified hemizygous *PLD1* mutations in the first, second, and third fetuses. The fourth fetus was heterozygous. In addition, the parents were heterozygous for *PLD1*. This case is based on three consecutive cases of homozygosity for the *PLD1* gene in the sibling cases and the fetuses with recurrent right ventricular valve dysplasia. This will elucidate the cause of recurrent congenital heart disease and intrauterine fetal death and may serve as an indicator for screening the next fetus. To date, homozygous mutations in *PLD1* that repeat three times in a row are not reported, only up to two times. The novelty of this report is that it was repeated three times, followed by a heterozygous live birth.

**Conclusions:**

This report is consistent with previous reports that mutations in *PLD1* cause right ventricular valve dysplasia. However, there have been few case reports of *PLD1* mutations, and we hope that this report will contribute to elucidate the causes of congenital heart disease, especially right ventricular valve dysplasia, and that the accumulation of such information will provide more detailed information on *PLD1* mutations in heart disease.

## Background

Congenital heart disease (CHD) occurs in approximately 1 in 100 cases [1]. Although sibling occurrence is high (3–9%), the majority of affected families comprise only one person with CHD [2]. Therefore, recurring CHD cases beyond three occurrences are exceptionally rare, and the majority of CHD cases are caused by multifactorial inheritance. Research to identify the causative genes is currently underway around the world, and there has been an increase the number of reports of cases in which specific genes have been found in the sibling population [3]. Phospholipase D (PLD) is an essential signaling enzyme in mammalian cells, including in cardiomyocytes. Additionally, PLD catalyzes the hydrolysis of phosphatidylcholine to produce phosphatidic acid, and *PLD1* is one of two of this mammalian PLD isozyme that has been cloned [4]. Recently, it has been reported that mutations in PDL1 are associated with dysplasia of the pulmonary artery valve [5], but there are few reports of such cases. In this report, we describe a case of triple fetal complicated cardiac malformation diagnosed as a PDL1 mutation. While this report represents a single case, it contributes to the understanding of the genetic etiology of CHD, particularly in relation to *PLD1* mutations.

## Case report

### Maternal background and first pregnancy

**Fig. 1:**
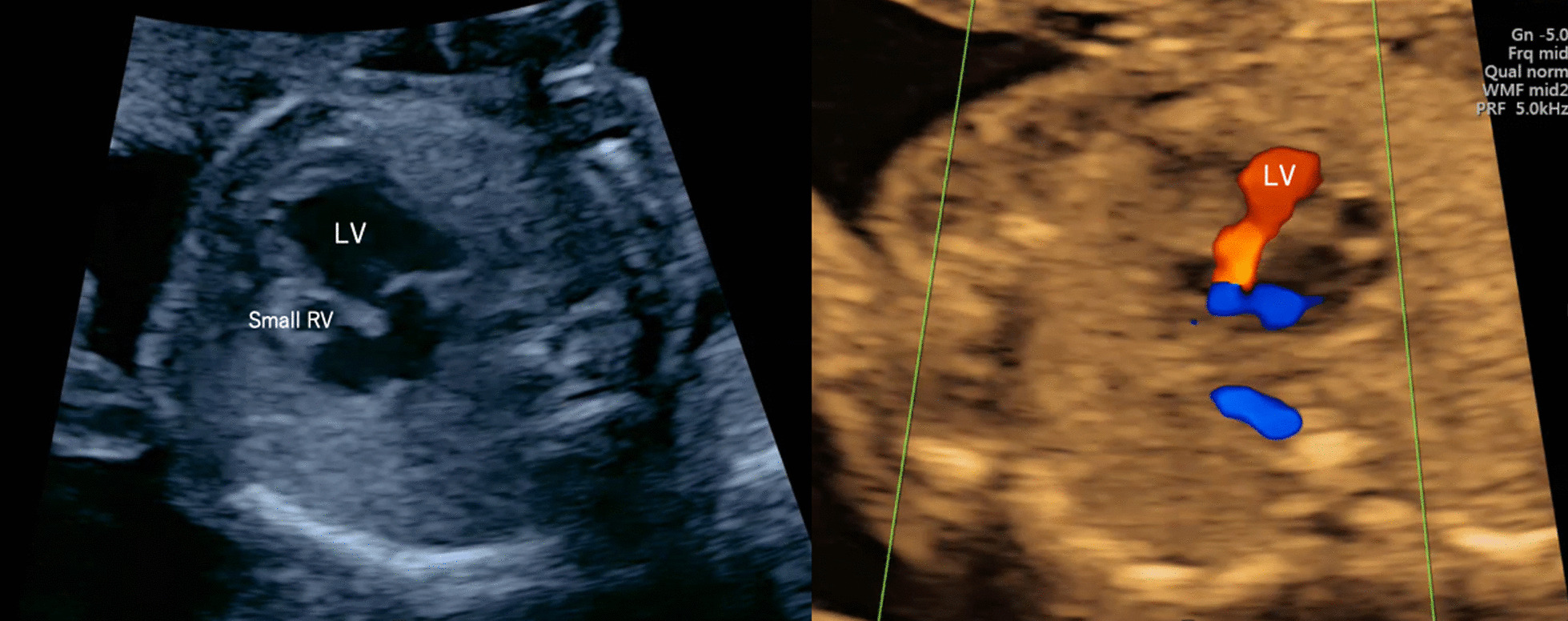
Fetal ultrasound images; 21 weeks and 1 day of gestation in the first pregnancy. Left image (B-mode): there was a left ventricular-type single ventricle. Right image (Color doppler): the pulmonary artery was closed and we observed the mitral regurgitation. LV, left ventricle; RV, right ventricle

A 32-year-old Japanese woman (gravida 1, para 0) underwent a prenatal checkup at a different hospital. Her past medical and family history is insignificant. She had no family with CHD. Her first pregnancy was spontaneous, and she had an antenatal checkup at 20 weeks and 6 days of gestation; the four-chamber view was unclear on fetal cardiac screening at the previous clinic and the patient was referred to our hospital at 21 weeks and 1 day of gestation. A results of a detailed ultrasound examination at our hospital showed an estimated fetal weight of 312 g [−1.3 standard deviation (SD)], a maximum vertical pocket (MVP) of 5.5 cm. We then diagnosed a diagnosis of ductus arteriosus-dependent left ventricular-type single ventricle and pulmonary artery obstruction. (Fig. 1). No significant complications were observed. Artificial abortion using Gemeprost was performed at 21 weeks and 5 days of gestation. The baby weighed 416 g at birth with no apparent malformations. No chromosomal examinations or pathological autopsies were performed.

### Second pregnancy

**Fig. 2:**
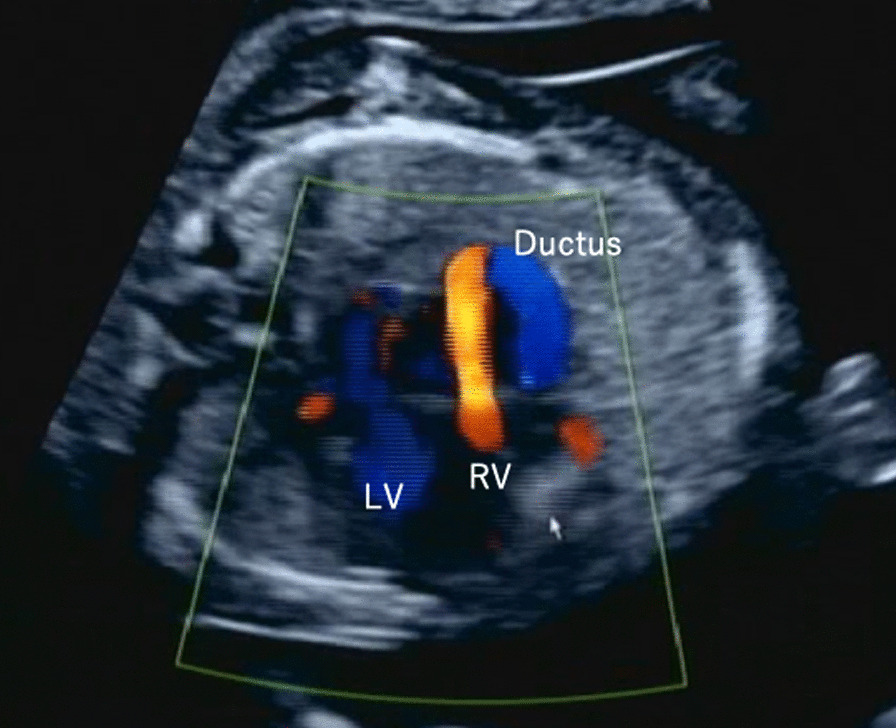
Fetal ultrasound images; 13 weeks and 3 days of gestation in the second pregnancy. A close examination of the heart revealed cardiac enlargement with a cardiothoracic area ratio (CTAR) > 35% and no pulmonary artery valve. The ductus arteriosus was retrograde. LV, left ventricle; RV, right ventricle

The second pregnancy, at the age of 33 years, was achieved by artificial insemination of the husband (AIH), and the patient visited our clinic at 9 weeks and 0 days of gestation. The cephalic length was 23.7 mm (9 weeks and 3 days of gestation), and the fetal heartbeat was confirmed. An ultrasound examination at 13 weeks and 3 days of gestation revealed a large transverse diameter of 21.8 mm (equivalent to 12 weeks and 5 days of gestation) and a cephalic length of 73.9 mm (equivalent to 13 weeks and 3 days of gestation). A close examination of the heart revealed cardiac enlargement with a cardiothoracic area ratio (CTAR) > 35% and no pulmonary artery valve. Additionally, poor right ventricular motion and severe tricuspid regurgitation were observed. The ductus arteriosus was retrograde, and pulmonary atresia with intact ventricular septum (PA/IVS) or Ebstein anomaly was suspected (Fig. 2). At the next visit (14 weeks and 3 days of gestation), fetal ultrasonography confirmed intrauterine fetal death. At the time of confirmation, the length of the cephalic cavity was 77.5 mm (equivalent to 14 weeks and 6 days of gestation), and delivery was performed 5 days later using gemeprost vaginal contraceptives. The baby was born weighing 30 g and measuring 10.5 cm in height. No apparent malformations were observed. A chromosomal examination was requested, and a placental villus was submitted, which showed a normal karyotype of 46, XX.

### Third pregnancy

**Fig. 3:**
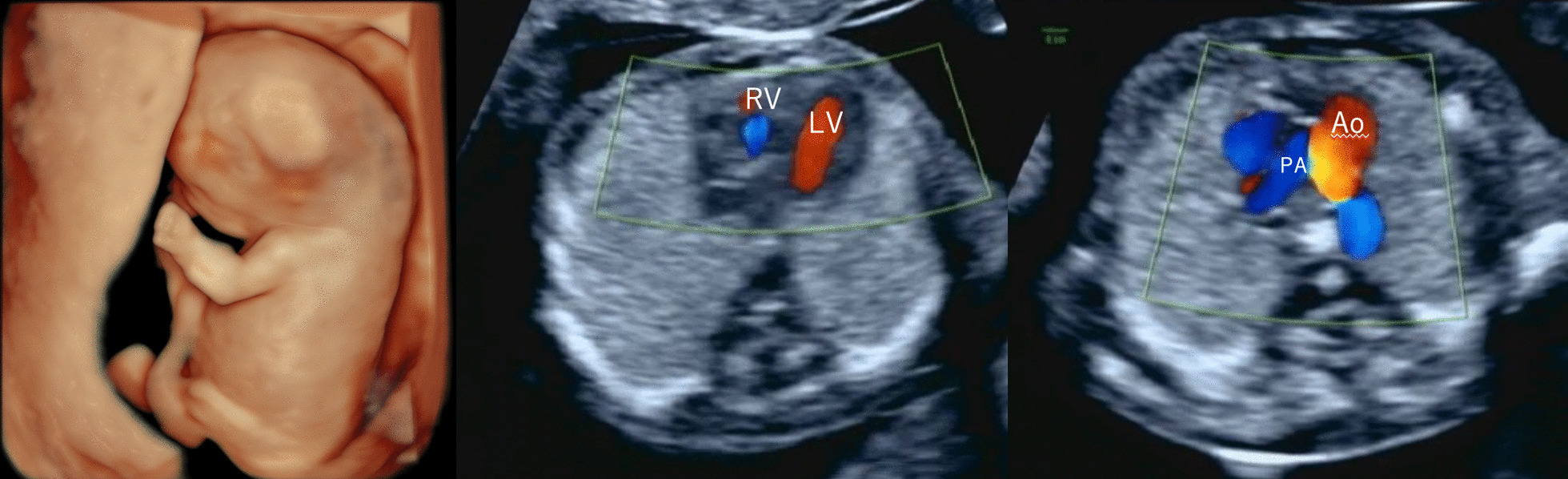
Fetal ultrasound images; 11 weeks and 5 days of gestation in the third pregnancy. Fetal ultrasonography showed mild fetal hydrops (the left image), moderate tricuspid valve regurgitation (the central image), and decreased right ventricular contractility (the right image)

**Fig. 4:**
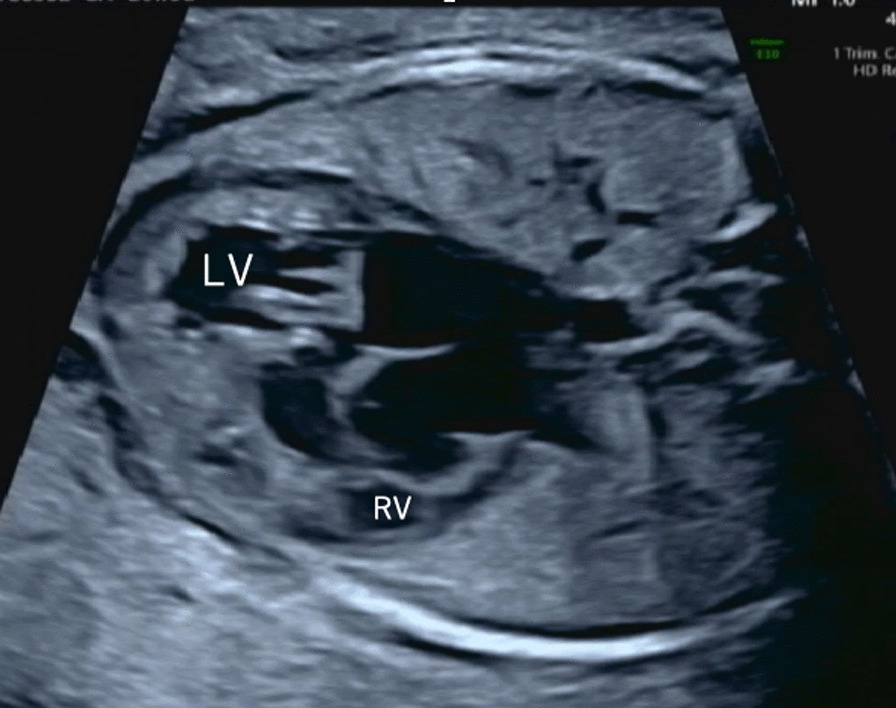
Fetal ultrasound images; 26 weeks and 5 days of gestation in the third pregnancy. The CTAR was 50% and there was pericardial effusion, thoracoabdominal effusion, decreased left ventricular contractility, and a decreased cardiovascular profile score of 4 points

A third pregnancy was established by in vitro fertilization, and the patient visited our clinic at 11 weeks and 5 days of gestation. Fetal ultrasonography on the same day showed mild fetal hydrops, moderate tricuspid valve regurgitation, and decreased right ventricular contractility (Fig. 3). At 13 weeks and 5 days of gestation, the fetal hydrops showed a mild trend toward resolution; however, tricuspid valve regurgitation was more than moderate and worsened, and right ventricular contractility continued to decrease. Therefore, pulmonary artery stenosis was suspected. A fetal cardiac examination by a pediatric cardiologist at 16 weeks and 5 days of gestation revealed a single left ventricle, a trace right ventricle, PA/IVS, and suspected cardiomyopathy. We performed amniocentesis at 17 weeks and 2 days of gestation, and the results showed normal karyotypes. After informed consent was obtained from the amniotic fluid test results, the parents wished to continue their pregnancy. Thereafter, the patient was closely monitored every week. At 26 weeks and 4 days of gestation, the estimated fetal weight was 1032 g (+0.3 SD), the amniotic fluid index was 15.1 cm, and the resistive index of the umbilical artery blood flow was 0.63 with no regurgitation. At 26 weeks and 5 days of gestation, a pediatric cardiologist performed fetal ultrasound, which revealed a CTAR of 50%, pericardial effusion, thoracoabdominal effusion, decreased left ventricular contractility, and a decreased cardiovascular profile score (CVPS) of 4 points (Fig. 4). Based on her current cardiac function and estimated fetal weight, we judged that postdelivery intervention would be difficult. Intrauterine fetal death was confirmed at the next visit at 27 weeks and 5 days of gestation, and delivery was performed 3 days later using a gemeprost vaginal suppository. The baby weighed 1244 g at birth with no obvious external malformations. A pathological autopsy was performed. The heart showed traces of the pulmonary artery from the right ventricle but no evidence of pulmonary valve traffic. The left ventricle was enlarged with thinning of the left ventricular wall, and the aorta was observed in the left ventricle. The patient was diagnosed with PA/IVS and cardiomyopathy. After consultation with the parents, the umbilical cords from the first to the third child were subjected to genetic analyses.

### Fourth pregnancy

The fourth pregnancy was established by in vitro fertilization at the age of 34. An ultrasound examination in the first trimester at 12 weeks and 5 days of gestation showed findings suggestive of nasal bone defects; however, no other abnormal findings were noted. Fetal ultrasonography performed at 15 weeks and 5 days of gestation revealed no obvious cardiac abnormalities. The mother requested an amniotic fluid examination, and after explaining the risks, an amniotic fluid examination was performed at 16 weeks and 4 days of gestation. Amniotic fluid test results showed a normal karyotype. At 26 weeks and 5 days of gestation, the midterm ultrasound examination was clear, and the estimated fetal weight was 921 g (−0.5 SD) with an amniotic fluid index (AFI) of 11.0 cm. The patient was transferred to another hospital, and vaginal delivery was performed at 38 weeks and 4 days of gestation. The baby was born weighing 3.128 g, measuring 49 cm, and was discharged without any abnormalities.

### Genetic information

We submitted the first and second fetuses’ umbilical cord, the third fetus’ placenta, and the fourth fetus’ blood to genetic testing using whole exome analysis with next generation sequencing. Genetic analysis identified a homozygous *PLD1* mutation in the analysis of the umbilical cords of the first, second, and third children. The fourth child was heterozygous. The parents were also found to be heterozygous. Based on the disease status, the *PLD1* mutation was confirmed as a match, and the results were disclosed by a physician in the genetic medicine department.

## Discussion

In this case, we found the pulmonary valve dysplasia was found in the first, second, and third pregnancy. Moreover, both parents were heterozygous for *PLD1*, and the three siblings were homozygous for *PLD1*. The fourth child was also heterozygous. No chromosomal test was performed the first time, but the second and third times, the tests showed normal karyotypes.

Regarding genetic involvement in CHD, about 80% of cases are multifactorial in onset, resulting from a combination of genetic, environmental, and other factors. The remaining 20% are due to chromosomal abnormalities, Mendelian syndromes, nonsyndromic single-gene disorders, and teratogens [6]. Approximately 400 genes are thought to be involved in the pathogenesis of CHD. Mutations in these genes can interfere with transcription factors, cell signaling, and chromatin modifications leading to structural abnormalities and dysfunction of the heart [7]. However, the cause of 60% of CHD is unknown, and research on the genetic etiology of CHD is ongoing [6].

Children born into families with preexisting CHD are at increased risk for developing CHD, with a 3–9% rate of sibling CHD [8]. In the Norwegian CVDNO project [3], 4.1% of children of identical parents with a sibling with CHD had CHD, compared with 1.1% of children without CHD [adjusted relative risk (RR) 3.6; 95% confidence interval (CI) 3.1–4.1]. Concerning gender, the RR was 14.0 (95% CI 10.6–18.6) for same-sex twins and 11.9 (95% CI 7.1–19.9) for opposite-sex twins. For half-siblings, the RR was 1.5 (95% CI 0.8–2.8). With respect to severity, for severe CHD, the RR was 6.9 (95% CI 4.9–9.8) for sibling incidence with identical parents. This means that families with CHD should carefully screened before having the next fetus [6].

Brodwall *et al*. also reported that in 50% of pairs with recurrent CHD, siblings had similar types of CHD [3]. Sabrina *et al*. aimed to determine the pattern of CHD recurrence within families [8], calculated the concordance rates for 41 types of CHD, and found a high degree of variability. They studied 1640 pairs of concordant and discordant lesions observed among affected families and identified 178 significant intrafamilial concordant pairs but no other concordant pairs. In other words, different types of CHD are more likely to cause intrafamilial onset, suggesting the influence of an underlying developmental mechanism. Subsequently, they investigated whether genes were matched even when the type of pathogenesis was mismatched and analyzed susceptibility genes in humans and mice, finding that 19% and 20% of the pairs were matched within families, respectively. Familial-onset CHD is most likely caused by overlapping susceptibility genes [8].

The risk of recurrence is reported to be higher in first-degree relatives compared with second- and third-degree relatives [9]. Also, the familial occurrence is more common in ductus arteriosus, pulmonary artery stenosis, ventricular septal defect, and tetralogy of Fallot (TOF) [10, 11]. The familial incidence of TOF is as high as 2.5–3% [10]. The involvement of NKX2.5 [12], JAGGED1 [13], and FOG2 [14] as causative genes has been demonstrated. The recurrence rate of TGA is 1.8%, and many genes are known to be involved [15]. Intrafamilial recurrence is also common in left ventricular hypoplasia, and NKX2.5 and NOTCH1 are known to be involved. However, intrafamilial CHD has unique and atypical recurrence patterns. Genes may cause CHD to appear in one member of the family but result in a normal heart of a relative [14]. Therefore, the inheritance mechanism remains unknown and requires further elucidation through genetic analysis.

Regarding the *PLD1* mutation, the *PLD1* gene encodes a phospholipase specific for phosphatidylcholine, which catalyzes the hydrolysis of phosphatidylcholine to produce phosphatidic acid and choline. For cardiac valve dysplasia, CVDP1 is caused by homozygous or compound heterozygous mutations in the *PLD1* gene at 3q26 [16]. For *PLD1* mutations, in 2021, Najim *et al*. [5] identified 30 patients with *PLD1* mutations and congenital heart valve defects as the main symptoms using whole exome sequencing and GeneMatcher. Missense mutants in PDL1 are abundant in regions of the protein that are critical for catalytic activity, and most *PLD1* mutant proteins show strongly reduced enzymatic activity. They also concluded that the inhibition of *PLD1* reduces endothelial–mesenchymal transition, an early step in valve formation, leading to an understanding of the disease mechanisms associated with the loss of *PLD1* function. Naiim *et al*. also noted the following regarding *PLD1* loss of function [2]: in patients with *PLD1* loss of function, structural defects in the atrioventricular valve were a major problem. The embryonic heart undergoes a pivotal early stage of valve formation when a subpopulation of endocardial cells overlapping the paracardial cushion undergo endothelial–mesenchymal transition (endo MT) [5]. These mesenchymal cells cause extracellular matrix remodeling and contribute to valve and septal wall development. Furthermore, in vitro experiments in chickens have demonstrated that *PLD1* function is involved in endo MT [4].

In addition, Ta-Shma *et al*. reported in 2017 [16] that the hearts of *PLD1* knockout mice showed marked tricuspid regurgitation, right atrial enlargement, increased pulmonary artery valve stenosis and flow velocity, and pulmonary artery valve thickening.

In light of the non-genetic aspects, the causes of CHD are largely unknown, with only about 15% of cases having an identified genetic cause [17]. Unraveling the complex relationship between genetics and environmental exposures. The complex relationship between genetics and environmental burden is important in understanding the multifactorial nature of CHD. Alcohol and illicit drug consumption and tobacco use have been widely studied as potential causal factors of CHD [17–19]. However, this literature has been largely inconsistent, with most positive associations being moderate at best. Dose–response relationships have been noted for tobacco and marijuana use, alcohol consumption, combined alcohol and tobacco use, and associations found only when both parents smoke [18–20]. Boyd *et al*. said that to make significant progress in the development of this model, continued progress in the exome field and the establishment of a bank of exome data from epidemiological studies are needed [17]. Moreover, cardiac responses to several genetic risks have been identified through transcriptional analysis of model organisms carrying human CHD mutations [21]. There are numerous environmental risk factors for CHD, including pesticides and therapeutic agents, and some of these are known to target specific genes. The molecular response of the heart to environmental teratogens is largely uncharacterized, but the transcriptional profile of zebrafish exposed to retinoic acid has been characterized [21].

 In this report, both parents were heterozygous for *PLD1*, and the child was homozygous for the *PLD1* mutation in three siblings. The pulmonary artery closure in the first and second pregnancies and the pulmonary artery stenosis in the third pregnancy were consistent with the dysplastic pulmonary valve described in Lahrouchi [5] and other reports. We carefully communicated this with the counselor present and the clinical geneticist. Both parents were told about the results and were satisfied with the series of fetuses. We are now considering whether we can perform preimplantation genetic testing for monogentic/single-gene diseases (PGT-M) when they wish to have another child.

This case has a limitation; that is, it is a very small report with a sample size of one case. However, reports of CHD in the *PLD1* gene are rare, so hopefully this report will lead to a better understanding of the genetic involvement of CHD in the future.

## Conclusion

A few reports on *PLD1* mutations have been published. In this report, three cases of repeated CHD are described. The fourth child was heterozygous and had a normal heart. From previous reports, the *PLD1* mutation with cardiac disease is only homozygous, which is consistent with our report. Future cohort studies are warranted to determine the proportion of children born with normal hearts even when both parents are heterozygous for *PLD1*. We hope that this report will help to elucidate the causes of repeated CHD, particularly for genetic counseling for right ventricular valve dysplasia, and that the accumulation of such information will lead to more detailed information on *PLD1* mutations in heart disease.

## Data Availability

Not applicable.

## Abbreviations

CHD: Congenital heart disease
MVP: Maximum vertical pocket
AIH: Artificial insemination of the husband
AFI: Amniotic fluid index
PA/IVS: Pulmonary atresia with intact ventricular septum
CTAR: Cardiothoracic area ratio
CVPS: Cardiovascular profile score
TOF: Tetralogy of Fallot
RR: Relative risk
CI: Confidence interval

## References

[1] Mahler GJ, Butcher JT (2011). Cardiac developmental toxicity. Birth Defects Res C Embryo Today.

[2] Oyen N, Poulsen G, Boyd HA (2009). Recurrence of congenital heart defects in families. Circulation.

[3] Brodwall K, Greve G, Leirgul E, Tell GS, Vollset SE, Oyen N (2017). Recurrence of congenital heart defects among siblings—a nationwide study. Am J Med Genet A.

[4] Tappia PS, Dent MR, Dhalla NS (2006). Oxidative stress and redox regulation of phospholipase D in myocardial disease. Free Radic Biol Med.

[5] Lahrouchi N, Postma AV, Salazar CM (2021). Biallelic loss-of-function variants in PLD1 cause congenital right-sided cardiac valve defects and neonatal cardiomyopathy. J Clin Investig.

[6] Blue GM, Kirk EP, Sholler GF, Harvey RP, Winlaw DS (2012). Congenital heart disease: current knowledge about causes and inheritance. Med J Aust.

[7] Williams K, Carson J, Lo C (2019). Genetics of congenital heart disease. Biomolecules.

[8] Ellesoe SG, Workman CT, Bouvagnet P (2018). Familial co-occurrence of congenital heart defects follows distinct patterns. Eur Heart J.

[9] Peyvandi S, Ingall E, Woyciechowski S, Garbarini J, Mitchell LE, Goldmuntz E (2014). Risk of congenital heart disease in relatives of probands with conotruncal cardiac defects: an evaluation of 1,620 families. Am J Med Genet A.

[10] Calcagni G, Digilio MC, Sarkozy A, Dallapiccola B, Marino B (2007). Familial recurrence of congenital heart disease: an overview and review of the literature. Eur J Pediatr.

[11] Chin-Yee NJ, Costain G, Swaby JA, Silversides CK, Bassett AS (2014). Reproductive fitness and genetic transmission of tetralogy of Fallot in the molecular age. Circ Cardiovasc Genet.

[12] Goldmuntz E, Geiger E, Benson DW (2001). NKX2.5 mutations in patients with tetralogy of fallot. Circulation.

[13] Eldadah ZA, Hamosh A, Biery NJ (2001). Familial tetralogy of fallot caused by mutation in the jagged1 gene. Hum Mol Genet.

[14] Pizzuti A, Sarkozy A, Newton AL (2003). Mutations of ZFPM2/FOG2 gene in sporadic cases of tetralogy of Fallot. Hum Mutat.

[15] Digilio MC, Casey B, Toscano A (2001). Complete transposition of the great arteries: patterns of congenital heart disease in familial precurrence. Circulation.

[16] Ta-Shma A, Zhang K, Salimova E (2017). Congenital valvular defects associated with deleterious mutations in the PLD1 gene. J Med Genet.

[17] Boyd R, McMullen H, Beqaj H, Kalfa D (2022). Environmental exposures and congenital heart disease. Pediatrics.

[18] Alverson CJ, Strickland MJ, Gilboa SM, Correa A (2011). Maternal smoking and congenital heart defects in the Baltimore-Washington Infant Study. Pediatrics.

[19] Matefa WA, Nelson DB, Kroelinger CD (2012). The association between maternal alcohol use and smoking in early pregnancy and congenital cardiac defects. J Womens Health.

[20] Wiliams LJ, Correa A, Rasmussen S (2004). Maternal lifestyle factors and risk for ventricular septal defects. Birth Defects Res A Clin Mol Teratol.

[21] Lage K, Greenway SC, Rosenfeld JA, Wakimoto H, Gorham JM, Segre AV (2012). Genetic and environmental risk factors in congenital heart disease functionally converge in protein networks driving heart development. Proc Natl Acad Sci.

